# A Novel Method of Coupling In Situ Time-Resolved FTIR and Microwave Irradiation: Application to the Monitoring of Quinoxaline Derivatives Synthesis

**DOI:** 10.3390/molecules30193875

**Published:** 2025-09-25

**Authors:** Alina Cherniienko, Kacper Kossakowski, Lucjusz Zaprutko, Roman Lesyk, Dorota Olender, Anna Pawełczyk

**Affiliations:** 1 Chair and Department of Organic Chemistry, Poznan University of Medical Sciences, 61-701 Poznan, Poland; kacper.kossakowski@student.ump.edu.pl (K.K.); zaprutko@ump.edu.pl (L.Z.); dolender@ump.edu.pl (D.O.); apaw@ump.edu.pl (A.P.); 2 Doctoral School, Poznan University of Medical Sciences, 61-701 Poznan, Poland; 3 Department of Pharmaceutical, Organic and Bioorganic Chemistry, Danylo Halytsky Lviv National Medical University, 79-010 Lviv, Ukraine; dr_r_lesyk@org.lviv.net; 4 Department of Biotechnology and Cell Biology, University of Information Technology and Management in Rzeszow, 35-225 Rzeszow, Poland

**Keywords:** in situ FTIR, time-resolved FTIR, optimisation, nitrogen heterocycles, quinoxaline, pyrazine, N-heterocyclisation, microwave-assisted synthesis

## Abstract

Traditional synthetic methods, often limited by inefficiency, are increasingly being replaced by sustainable alternatives. This study presents a green approach combining microwave irradiation with in situ FTIR spectroscopy for real-time monitoring and optimising nitrogen-heterocycle synthesis, focusing on quinoxalines. Although both microwave-assisted synthesis and time-resolved FTIR are established techniques, their combined application remains underexplored, limiting their synergistic potential. The methodology was applied to synthesising 2,3-diphenylquinoxaline, a compound of interest in medicinal chemistry. Optimisation of the condensation between benzil and 1,2-phenylenediamine was achieved by exploiting the accelerated kinetics of microwave irradiation and continuous monitoring via in situ FTIR. Three catalytic systems were evaluated—hydrochloric acid (Brønsted acid), Montmorillonite K10 (heterogeneous catalyst), and molecular iodine (halogen/Lewis acid)—alongside a range of solvents, including ethanol, methanol, water, acetonitrile, ethyl acetate, dimethyl sulfoxide, and dichloromethane. Iodine proved to be the most efficient catalyst, while acetonitrile and ethyl acetate provided the most effective solvent systems. This integrated monitoring strategy reduces reliance on trial-and-error optimisation and establishes a streamlined, scalable, and efficient protocol. The dual-technique approach highlights a versatile pathway for advancing green synthetic methodologies with applications across the chemical and pharmaceutical industries.

## 1. Introduction

The field of medicinal chemistry faces significant challenges in the efficient synthesis of active compounds. Traditional methods are often time-consuming and costly, primarily due to the extensive trial-and-error synthesis route. In response to these challenges, there is a growing demand for novel advanced analytical techniques. This demand has spurred the development of real-time methods that provide detailed insights into ongoing chemical reactions, enabling more precise and efficient synthesis. These advancements not only reduce the time and cost burdens associated with unoptimised chemical processes but also accelerate the discovery of new therapeutic agents [1].

In situ Fourier transform infrared (FTIR) spectroscopy has become a crucial tool for non-invasive monitoring and characterisation of molecular transformations. This technique offers a versatile approach to investigating various chemical reactions, allowing researchers to observe reaction mechanisms, optimise conditions, and refine reaction pathways. In multiple industries, particularly the chemical and pharmaceutical sectors, FTIR spectroscopy provides a unique capability to detect and analyse complex chemicals while generating minimal chemical waste and reducing the need for extensive sample preparation or harmful reagents. In situ FTIR spectroscopy operates on principles similar to traditional FTIR, where an infrared light beam travels through a sample, and the absorbed energy creates a unique spectrum corresponding to the molecular vibrations within the sample. Unlike ex situ methods, which require removing and analysing samples outside their original environment, in situ FTIR allows direct, continuous monitoring of chemical reactions within the reaction vessel. This capability is particularly valuable, as it enables the investigation of reaction mechanisms and the identification of transient intermediates. However, the procedure requires specialised equipment and can be experimentally complex, posing challenges regarding accurate sample handling and setup. Overall, in situ FTIR spectroscopy offers a powerful means to advance the field of medicinal chemistry and other areas by providing a deeper understanding of complex reaction processes and intermediate species, contributing to the development of more efficient and sustainable chemical processes [2,3,4,5].

On the other hand, microwave (MW) irradiation is a powerful driver of chemical reactions and offers several advantages over classical conditions, making it an efficient and attractive alternative. MW irradiation provides rapid and uniform heating, significantly reducing reaction times compared to conventional methods. It is also more energy-efficient, as it directly couples with the reactants, minimising energy loss to the surrounding environment. The enhanced energy absorption of polar molecules often results in higher reaction rates and improved yields [6,7,8].

While both in situ FTIR spectroscopy and MW irradiation are well-established techniques, their combined application remains underexplored and insufficiently documented in the literature. An earlier study by Leadbeater and team [9] reported the first attempts to integrate microwave technology with in situ IR monitoring, though this approach was demonstrated only in a limited fashion. Building on this foundation, the present study seeks to expand the scope of this combined methodology by applying it more extensively—not only for monitoring individual reaction conditions, but also for systematically comparing solvents, catalysts, and related parameters to optimise widely used synthetic procedures relevant to large-scale applications in the chemical and pharmaceutical industries. By demonstrating how this coupled technique can be routinely employed to evaluate and control reactions in real-time, we highlight its potential for minimising time, energy consumption, and resource usage while maximising efficiency. The ability to monitor and adjust reaction conditions instantaneously reduces dependency on post-synthesis analyses, streamlining production and enhancing reproducibility. As industries seek more efficacious, cost-effective manufacturing solutions, integrating in situ FTIR with MW irradiation could accelerate reaction development and ensure high-yield, scalable, and environmentally friendly processes.

**Figure 1:**
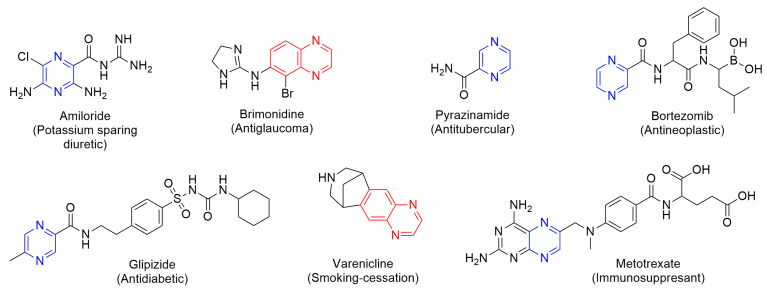
Examples of drugs containing 1,4-diazine scaffold—pyrazines (blue) and quinoxalines (red) [11,19].

Among the leading types of valued medicinal structures, heterocyclic systems are frequently obtained and undoubtedly require optimisation of methods for their synthesis. According to statistics, nitrogen-containing heterocyclic moieties (N-heterocycles) are present in more than 75% of medications approved by the FDA and are now on the market [10]. Many new N-heterocycles have been designed recently, among which 1,4-diazines, such as pyrazines and quinoxalines, are crucial structural constituents of many novel and future medications and bioactive substances (Figure 1) [11,12,13,14,15,16,17,18].

**Figure 2:**
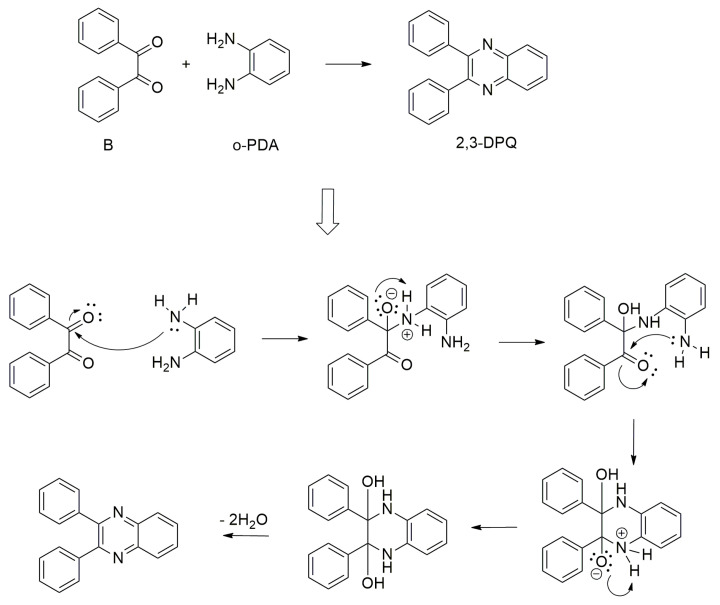
Synthesis of 2,3−diphenylquinoxaline and proposed mechanism of reaction.

Quinoxalines are rarely found in nature, although they can be produced in the laboratory and utilised in various insecticides, herbicides, dyes, antibiotics, anti-cancers, antivirals and other therapeutic molecules [11,20,21,22]. As a result, the synthesis of a 2,3-diphenylquinoxaline (2,3-DPQ) system from 1,2-phenylenediamine (o-PDA) and benzil (B) (Figure 2) was chosen for analysis using the technique we are developing. The quinoxaline scaffold was selected for analysis due to its extensive documentation in the literature, especially as the first step in synthesising more complicated quinoxaline structures [23,24]. This scaffold and other N-heterocycles targeted for future investigation have demonstrated favourable reactivity under microwave conditions [25,26,27], making it a compelling candidate for accelerated reaction kinetics and improved synthetic efficiency.

## 2. Results and Discussion

**Figure 3:**
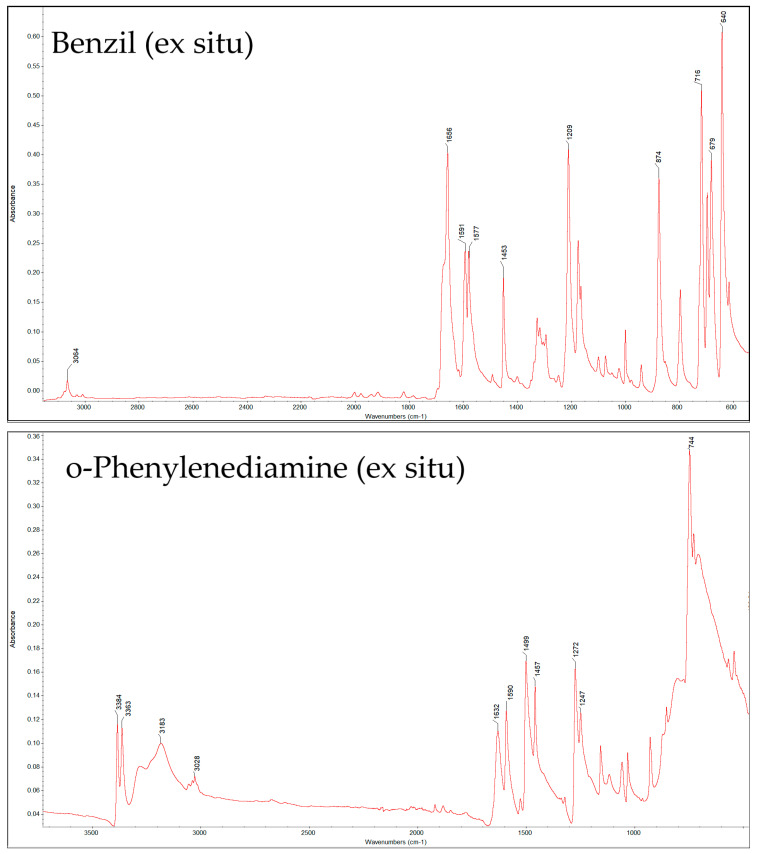

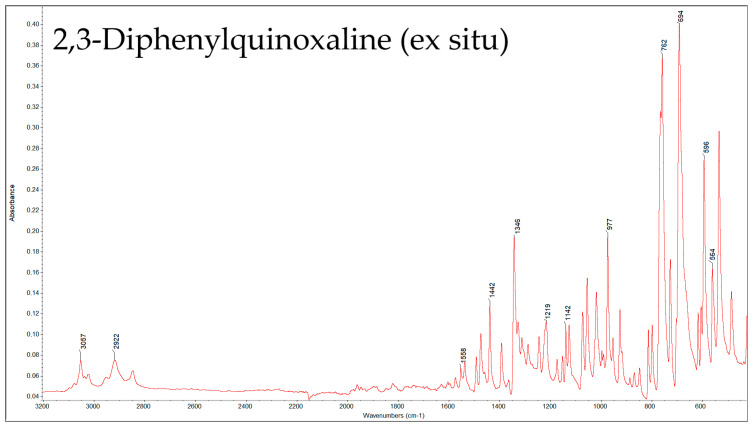
Ex situ FTIR spectrum of: Benzil, o−Phenylenediamine and 2,3−Diphenylquinoxaline.

This study evaluated the reaction time in different solvents and conditions and compared them to determine the optimal conditions for a safe and efficient 2,3-DPQ synthesis. The starting materials, including B and o-PDA, and crude and purified products, were characterised in their solid state using ex situ FTIR spectroscopy and via in situ FTIR. The results of the ex situ FTIR analysis are presented in a broad spectral overview (Figure 3), and the characteristic peaks for chemical structures of the reagents and products are summarised in Table 1.

**Figure 4:**
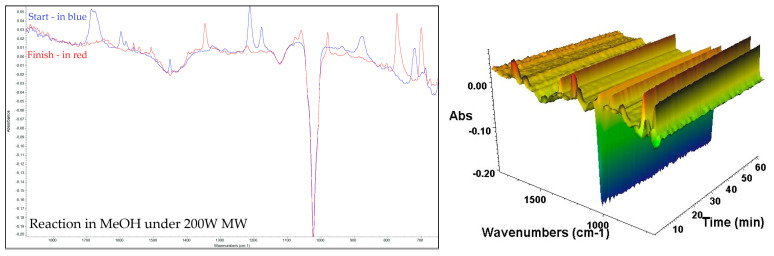
FTIR spectrum of: Benzil (blue)—in situ—in MeOH (10 mL); crude reaction mixture (red) at the finish of the reaction in MeOH (10 mL), and 3D Kinetics map of reaction.

The solvents employed for the condensation reaction included methanol (MeOH), ethanol (EtOH), methanol–water (7:3 ratio), dimethyl sulfoxide (DMSO), acetonitrile (MeCN), ethyl acetate (EtOAc), dichloromethane (DCM) and water. These solvents were selected based on their prior use in 2,3-DPQ synthesis as reported in the scientific literature [10,24]. The reactions were carried out under different conditions: under reflux and under microwave irradiation at 100 W and 200 W, all of which were monitored via in situ FTIR and thin-layer chromatography (TLC) throughout the process. Additionally, reactions were conducted at room temperature with stirring and analysed at different stages using ex situ FTIR and TLC. The processes were meticulously recorded and archived for subsequent analysis; also, the reactions were repeated numerous times in the same conditions to check and secure the accuracy of the results. For example, the reaction in MeOH at 200 W MW with concentrated HCl as the catalyst, shown in Figure 4, (includes the start and finish in situ FTIR spectra and 3D kinetics map of reaction), with characteristic peaks described, as illustrated in Table 2.

**Figure 5:**
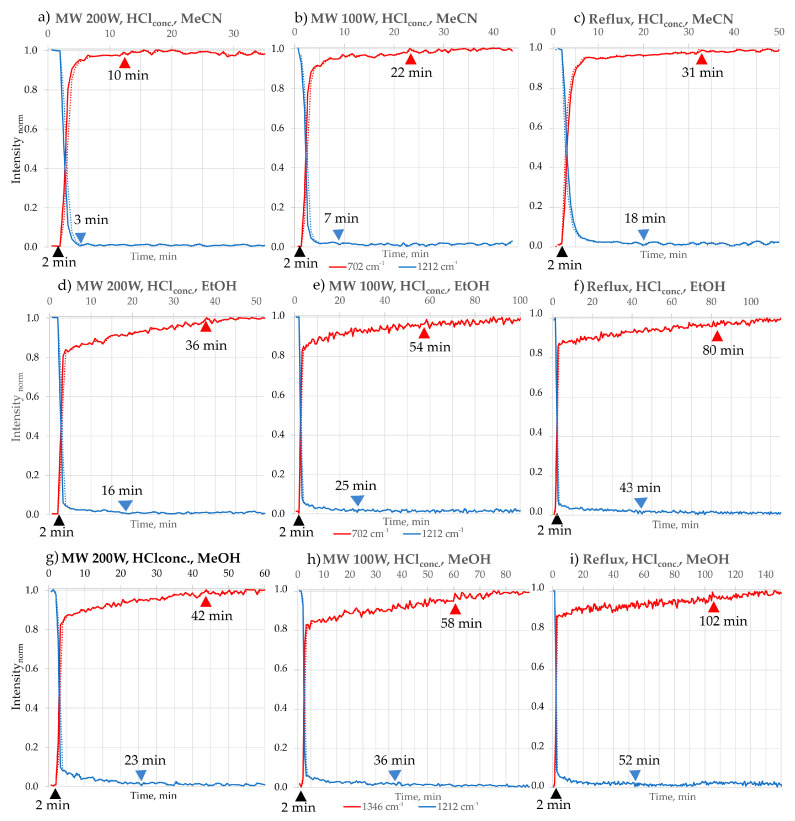
Intensity profiles of the 2,3-DPQ synthesis using concentrated HCl as catalyst in different solvents and conditions: MeCN: (**a**) MW 200 W: Peak intensity profile showing disappearance of substrate B (1211 cm^−1^, blue) and formation of 2,3−DPQ scaffold (703 cm^−1^, red). Approximate reaction completion: 10 min; (**b**) MW 100 W: Approximate reaction completion: 22 min; (**c**) Reflux: Approximate reaction completion: 31 min. 2. EtOH: (**d**) MW 200 W: Peak intensity profile showing disappearance of B (1211 cm^−1^, blue) and formation of 2,3-DPQ scaffold (770 cm^−1^, red). Approximate reaction completion: 36 min; (**e**) MW 100 W: Approximate reaction completion: 54 min; (**f**) Reflux: Approximate reaction completion: 80 min. 3. MeOH: (**g**) MW 200 W: Peak intensity profile showing disappearance of B (1211 cm^−1^, blue) and formation of 2,3-DPQ scaffold (1346 cm^−1^, red). Approximate reaction completion: 42 min; (**h**) MW 100 W: Approximate reaction completion: 58 min; (**i**) Reflux: Approximate reaction completion: 102 min. Symbols: ▲—time of o-PDA addition; ▲—B consumption time; ▲—2,3-DPQ forming stabilisation time.

**Figure 6:**
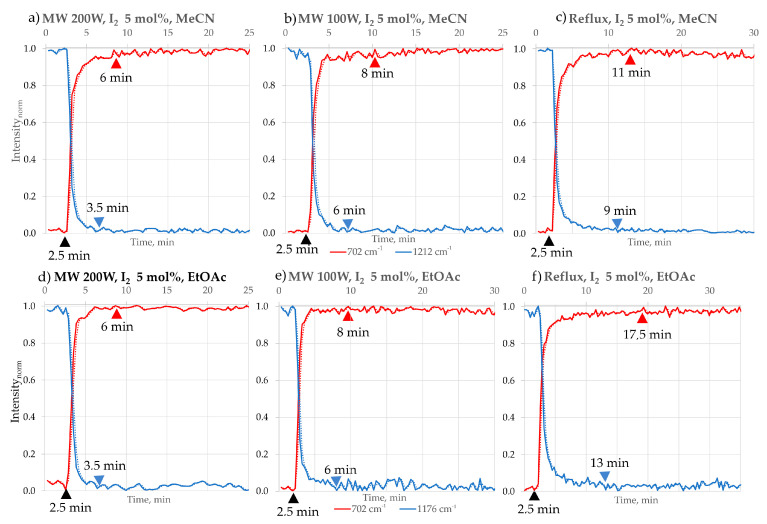
Intensity profiles of the 2,3−DPQ synthesis using I_2_ (5 mol%) as catalyst in different solvents and conditions: MeCN: (**a**) MW 200 W: Peak intensity profile showing disappearance of substrate B (1211 cm^−1^, blue) and formation of 2,3−DPQ scaffold (702 cm^−1^, red). Approximate reaction completion: 6 min; (**b**) MW 100 W: Approximate reaction completion: 8 min; (**c**) Reflux: Approximate reaction completion: 11 min. 2. EtOAc: (**d**) MW 200 W: Peak intensity profile showing disappearance of B (1176 cm^−1^, blue) and formation of 2,3−DPQ scaffold (702 cm^−1^, red). Approximate reaction completion: 6 min; (**e**) MW 100 W: Approximate reaction completion: 8 min; (**f**) Reflux: Approximate reaction completion: 17.5 min. Symbols: ▲—time of o-PDA addition; ▲—time of B consumption; ▲—stabilisation of 2,3-DPQ formation.

**Figure 7:**
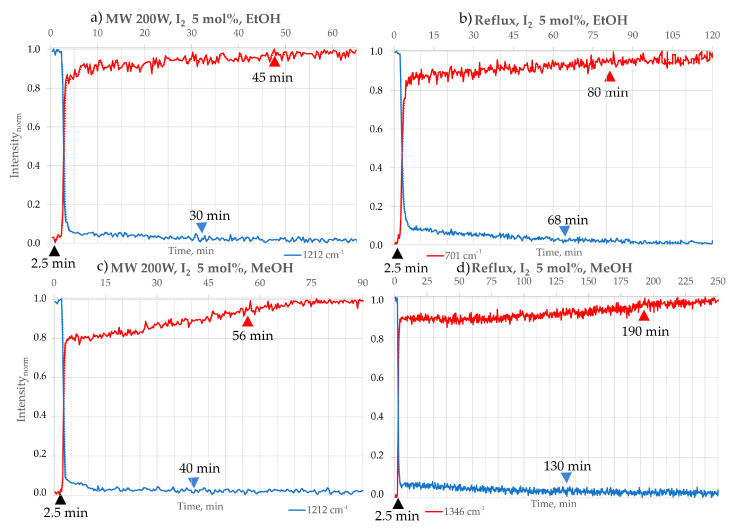
Intensity profiles of the 2,3−DPQ synthesis using I_2_ (5 mol%) as catalyst in different solvents and conditions: EtOH: (**a**) MW 200 W: Peak intensity profile showing disappearance of substrate B (1211 cm^−1^, blue) and formation of 2,3-DPQ scaffold (702 cm^−1^, red). Approximate reaction completion: 46 min; (**b**) Reflux: Approximate reaction completion: 80 min. 2. MeOH: (**c**) MW 200 W: Peak intensity profile showing disappearance of B (1211 cm^−1^, blue) and formation of 2,3−DPQ scaffold (1346 cm^−1^, red). Approximate reaction completion: 56 min; (**d**) Reflux: Approximate reaction completion: 190 min. Symbols: ▲—time of o-PDA addition; ▲—time of B consumption; ▲—stabilisation of 2,3−DPQ formation.

The progress of the reaction was assessed by creating and analysing chosen peak profiles. The term “FTIR peak profile” refers to various methods representing analysis results from data obtained from in situ FTIR measurements. In this study, we have chosen the “Peak Height of One Peak” profile, which represents the frequency location of a specific peak along with its baseline endpoints throughout the ongoing process. Also, for some peaks that may have been affected by interference from a broad peak caused by H_2_O, we used the “Peak Area of One Peak” profile. This approach calculates the corrected area of the specified peak for each spectrum, considering the frequency limits of the peak and the baseline endpoints. In Figure 5, Figure 6 and Figure 7, the chosen intensity profiles of the 2,3-DPQ synthesis are presented in concentrated HCl and I_2_ catalytic option. The reaction’s endpoint was determined by observing the stabilisation of peak intensity values. All intensity values were normalised using min–max normalisation to enable direct comparison between solvents and experimental conditions.

The FTIR peak profiles presented in the publication demonstrate the disappearance of the B characteristic peak at 1211 cm^−1^, corresponding to the C-C bending vibration of ketones, in each solvent system. However, the peak profiles for 2,3-DPQ formation varied depending on the solvent used, as some solvent molecules may overshadow specific absorption bands due to their structural properties. In MeCN, the characteristic peak at 703 cm^−1^ was selected, representing the C-H in-plane vibrations of the aromatic benzene ring. For EtOH, the chosen peak was at 770 cm^−1^, also attributed to C-H in-plane vibrations of the aromatic benzene ring. In MeOH, the peak at 1346 cm^−1^ was identified, corresponding to C-N/C-C (phenyl) vibrations.

By analysing the intensity profiles of chosen peaks (Figure 5, Figure 6 and Figure 7), we have been able to describe the approximate time of reaction completion, which is presented in Table 3. The results were double-checked by TLC. Comparisons were made between the conventional (room temperature and reflux) and microwave methods (100 and 200 W) in different reaction media (Table 3).

The tested N-heterocyclisation reaction with complete conversion occurs in solvents such as MeCN, EtOAc, EtOH and MeOH. The reactions carried out under microwave irradiation consistently afforded the shortest reaction time (Table 3) and the highest isolated by column chromatography yields, ranging from 99 to 95%, reflecting complete substrate conversion. The best results were obtained in MeCN and EtOAc, whereas slightly lower yields were observed in MeOH and EtOH. Reactions also proceeded efficiently, though with somewhat reduced yields at reflux conditions (95–90%) and room temperature with magnetic stirring (91–86%). Overall, all investigated conditions delivered high product yields, underscoring the robustness of the transformation. An increase in the reaction temperature—from room temperature (r.t.) to the boiling point (b.p., reflux) of the solvent used—causes a significant shortening of the reaction time in each solvent variant, consistent with the Arrhenius equation. In addition, an increase in the microwave irradiation power from 100 W to 200 W also translates into a further shortening of the reaction time.

The application of microwave (MW) methodologies is fully consistent with the principles of green chemistry, as they minimise waste generation and reduce energy consumption. In the context of organic compound synthesis, it has been demonstrated that performing reactions under green conditions facilitated by MW irradiation results in a yield increase of approximately 4–5% compared to conventional reflux and 6–9% compared to stirring at room temperature. Moreover, reaction rates were accelerated by up to three-fold under reflux conditions and up to twelve-fold at room temperature when MW irradiation was employed. Consequently, the substantial reduction in reaction times directly contributes to decreased total energy consumption and, in turn, to a lower overall environmental impact. These findings highlight that the use of MW irradiation offers a significant improvement in efficiency and sustainability over classical synthetic approaches.

For the two most favourable variants, such as for HCl and I_2_ catalyst with MeCN, the simple time reduction factor, for a quick comparison of the processes, is introduced and presented in Table 4.

The most significant reduction in the reaction time of B with o-PDA is observed with I_2_ as a catalyst. For a microwave power of 200 W, the reaction time is 6 min, while reducing the power by half extends the reaction time by more than 1.3 times (8 min). On the other hand, at the conventional heating of MeCN (b.p. 81 °C), the reaction time is extended by more than 2 times (11 min) compared to the optimal time at 200 W of MW in MeCN.

In the HCl catalyst, the observed upward trend in time is stronger. The reaction at 100 W of MW takes about 2 times longer (22 min) than at 200 W of MW (10 min), while at conventional heating, it takes almost 3 times longer (31 min) compared to the optimal time at 200 W in EtOH.

Finally, the reaction is fastest in microwave conditions (200 W), and I_2_ takes almost 1.7 times faster than with HCl, and nearly 3 times faster at 100 W and reflux conditions.

It is frequently anticipated that solvents with stronger polarity—shown by higher dielectric constants and loss factors—will interact with microwave radiation more efficiently in microwave-assisted synthesis, resulting in faster reaction rates through effective heating. Nevertheless, our experimental findings showed an intriguing departure from this prediction. Regarding lowering reaction completion time, MeCN and EtOAc— which have a lower dielectric constant than more polar solvents like water or MeOH—were the most successful solvents among those tested.

Rationale behind that might be that protic solvents (MeOH, EtOH) can donate hydrogen bonds to substrates and catalysts. The o-phenylenediamine nucleophile can be hydrogen-bonded to protic solvent, which reduces its availability to attack the carbonyl. Likewise, the carbonyl oxygens of benzil may form H-bonds with solvent, decreasing carbonyl electrophilicity. Aprotic solvents (MeCN, EtOAc) cannot donate H-bonds, so amines and carbonyls are “free” to react. For example, imine formation (amine + carbonyl) is generally slower in protic media because the solvent H-bonds stabilise both reactants and intermediate hydroxylammonium species, raising the activation energy. In summary, the lack of H-bond donation in MeCN/EtOAc keeps the reactants and catalysts more reactive, whereas MeOH/EtOH tend to “lock up” reactants in solvated shells. Boiling point differences also matter. MeOH boils at 65 °C, whereas EtOH (78 °C), EtOAc (77 °C) and MeCN (82 °C) boil near or above 77 °C. Thus, reflux in MeCN/EtOAc can reach higher temperatures than MeOH. The ~15–20 °C higher reflux temperature in MeCN/EtOAc accelerates the reaction kinetics (per Arrhenius) compared to MeOH reflux.

**Figure 8:**
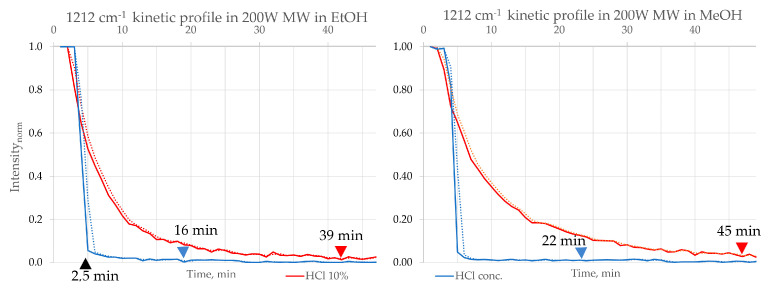
Intensity profiles of the B consumption (1212 cm^−1^) in the synthesis of 2,3−DPQ at MW 200 W using different HCl catalysts—concentrated HCl (blue) and 10%HCl (red). B consumption time: in EtOH: concentrated HCl—16 min; 10% aqueous HCl—39 min; in MeOH: concentrated HCl—22 min; 10% aqueous HCl—45 min. Symbols: ▲—time of o-PDA addition; ▲—time of B consumption with HCl conc. ▲—time of B consumption with HCl 10%.

Notably, some solvents and catalysts were deliberately excluded during the initial stages of data collection. The rationale behind this decision is as follows:

- MeOH–Water and Water: The inclusion of water led to prolonged reaction times compared to MeOH alone, because pure water or water-rich mixtures strongly hydrogen-bond to the o-phenylenediamine. Protic solvents stabilise the amine’s lone pairs (lowering its energy) and thus reduce its nucleophilicity. Furthermore, the presence of a broad O-H peak in the solvent at 1630 cm^−1^ interfered with the accurate assessment of the reaction, particularly in the obstruction of the 1684 cm^−1^ C=O carbonyl peak.
- DMSO: Observations made under MW and non-MW conditions unveiled the formation of undesirable side products only after the influence of MW. Additionally, the reaction failed to reach completion in all conditions, as indicated by the persistence of the B substrate, corroborated by TLC and IR analyses. By-products may occur because strong absorbers, such as DMSO, have high dielectric losses (above 14.00), causing them to heat rapidly in a microwave chamber. DMSO, in particular, decomposes into toxic substances at high temperatures, which are quickly reached under microwave irradiation. This decomposition can produce sulfur dioxide, formaldehyde, methyl mercaptan, dimethyl sulfide, dimethyl disulfide, and bis(methylthio)methane.
- DCM: Rapid crystallisation was observed in the reaction mixture. Immediate solid formation hindered proper mixing of the reagents, and the reaction did not easily proceed even upon prolonged heating. The precipitated crystals remained insoluble under the applied conditions, thereby preventing further monitoring of the progression of the transformation.
- The use of Montmorillonite K10 as a catalyst in molar concentrations ranging from 10 to 40 mol% proved inefficient. Although increasing the catalyst loading led to a reduction in reaction time, the improvement was limited—even at 200 W microwave power, 40 mol% of Montmorillonite K10 resulted in a reaction completion time of 80 min, compared to 130 min at 10 mol% in MeCN.
- The use of iodine (I_2_) at 10 mol% was found to be suboptimal compared to 5 mol% (Table 5), as the reaction reached completion more rapidly under the lower catalyst loading. Molecular iodine (I_2_) is well recognised as an efficient and sustainable catalyst in various organic transformations, including cycloadditions, Michael additions, esterifications, and heterocyclic syntheses. Its catalytic activity is primarily attributed to halogen bonding and its ability to act as a mild Lewis acid, thereby lowering activation barriers. In numerous reported protocols, small catalytic loadings (typically 5–10 mol%) can accelerate reactions and deliver excellent yields. However, the effect of iodine loading is known to be substrate- and reaction-dependent. For example, Zhang et al. reported that increasing I_2_ from 1 mol% to 5 mol% improved the yield of an annulation reaction to 86%, but further increase beyond 5 mol% resulted in no additional benefit [34]. In our system, we carefully repeated the reaction (up to five times under identical conditions to exclude experimental error) in MeOH, EtOH, and MeCN. We consistently observed that 5 mol% I_2_ afforded better reaction time than 10 mol%. We hypothesise that excess iodine may participate in undesired side processes. In particular, o-PDA, one of the key substrates, is prone to oxidation under oxidative conditions, and higher iodine loading may facilitate such competing pathways, lowering the overall yield. This observation is consistent with the data summarised in the table below:

- Also, the negative influence of water on reaction kinetics was observed by employing a 10% HCl solution as a catalyst, applied in the same proportion as concentrated HCl (Figure 8). A marked increase in reaction time was noted under various conditions when using the diluted HCl solution, highlighting the critical role of water in modulating reaction efficiency. In MeOH under 200 W, the reaction time increased from 22 min with concentrated HCl to 46 min with 10% HCl. For EtOH, under 200 W, the reaction time increased from 18 to 49 min. The reaction between B and o-PDA, forming 2,3-DPQ, provides an illustrative case study for examining the influence of water on acid-catalysed transformations. This reaction typically proceeds via a condensation mechanism, where removing water as a by-product is crucial for driving the equilibrium toward product formation. When water is present in the reaction medium, either as part of the solvent system or introduced through dilution of the catalyst, the equilibrium is disrupted, favouring the reactants and significantly reducing the reaction rate. Additionally, water molecules can compete with the reactants for active catalytic sites, thereby hindering the catalytic activity of HCl. These findings emphasise the importance of minimising water content in acid-catalysed reactions involving B and o-PDA, particularly when aiming for high efficiency and reduced reaction times.

The IR spectra served as a crucial analytical tool to confirm the formation of the desired product by identifying characteristic absorption bands corresponding to specific functional groups in both the substrate and the final product. This analysis also determined the most effective synthetic conditions for 2,3-DPQ, namely microwave irradiation in MeCN and EtOAc with iodine as the catalyst. Although several excluded solvents and catalysts are widely described in the scientific literature as promising options for analogous transformations, their systematic recreation and evaluation in this study—through IR spectroscopy and complementary analytical methods—revealed them to be inefficient and ultimately unsuitable for the present reaction. This discrepancy underscores the importance of experimental validation and demonstrates that literature precedent cannot always be directly extrapolated, particularly when optimising conditions for efficient synthesis.

Despite the advantages of integrating in situ FTIR spectroscopy with microwave irradiation, several methodological limitations must be acknowledged. First, using a diamond ATR probe restricts detection in certain spectral regions; however, this material is necessary due to its high resistance to chemical damage by organic solvents. Importantly, the most informative mid-infrared region relevant to organic transformations (1900–600 cm^−1^) remains fully accessible. A second limitation arises from solvent interference, as solvents are typically present in significant excess and their absorption bands can overlap with signals of interest. To mitigate this, solvent spectra are recorded as backgrounds for data analysis. While this procedure may partially mask specific spectral features, in most cases, only a limited region is affected, and alternative diagnostic bands can be employed to track the reaction pathway. For instance, in the case of methanol, strong background absorption prevents analysis in the 1000–1050 cm^−1^ range; nevertheless, other key bands remain suitable for monitoring, such as the disappearance of the benzil signal at 1212 cm^−1^ and the emergence of the 2,3-diphenylquinoxaline band at 1346 cm^−1^. These considerations highlight that while specific spectral windows may be compromised, reliable reaction monitoring is still achievable.

Alongside these limitations, the combined application of microwave-assisted synthesis with in situ FTIR monitoring offers several significant advantages. First, the methodology enables real-time, closed-vessel reaction (by closed vessel authors understand reaction flask connected to condenser, thermal and IR probes) profiling without the need for sampling, thereby avoiding cooling–heating artefacts and delays inherent to offline analysis. The ability to acquire densely sampled kinetic data provides high-resolution concentration-time profiles, accelerating optimisation. Compared to conventional trial-and-error methods, this approach has been shown to reduce reaction times (Table 3 and Table 4), minimise solvent consumption by eliminating redundant runs and solvent-intensive analyses such as HPLC, and lower overall energy input due to microwaves’ rapid and targeted heating. In addition, real-time monitoring allows immediate reaction termination at the desired conversion, thus improving selectivity and preventing unnecessary side-product formation.

With respect to scalability, this dual-technique approach is well aligned with current industrial process development trends, as microwave reactors and process-analytical technologies (PAT) are increasingly being adopted in large-scale chemical and pharmaceutical manufacturing. The inherent advantages—shorter reaction times, reduced solvent use, and improved energy efficiency—directly support sustainable industrial practices. Nevertheless, further pilot- and production-scale studies are required to address specific challenges such as optimising microwave penetration in larger vessels and adapting FTIR probes to continuous-flow or industrial batch reactors. These factors highlight the immediate benefits of the integrated approach and its promising potential for translation into large-scale applications.

## 3. Materials and Methods

### 3.1. Equipment

**Figure 9:**
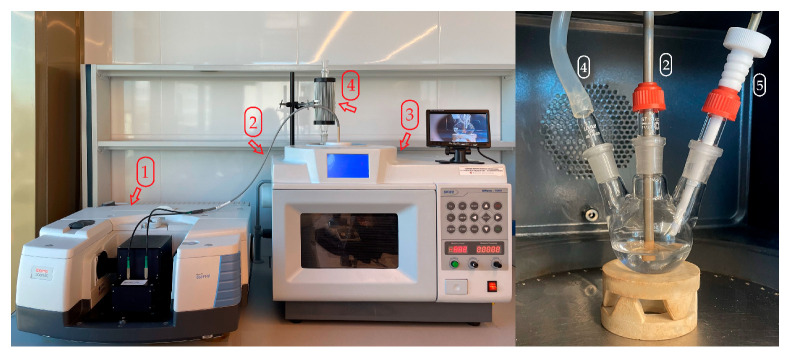
Synthetical-analytical system, consisting of an ATR-FTIR spectrometer (1) connected by a fibre optic probe (2) with multifunctional chemical reactor (3), equipped with condenser (4) and temperature probe (5).

The proposed research project used a multifunctional chemical reactor, the UWave-1000 by SINEO Microwave Chemistry Technology (Shanghai, China), to conduct the selected chemical reactions. This reactor can support chemical reactions using MW/UV/US agents, making it a versatile tool in the synthesis process, equipped with a multimode microwave generator (0–1000 W), an ultrasonic probe (26–28 kHz, 0–800 W), a UV lamp (λ = 365 nm, 300 W), contact and non-contact thermometer, magnetic stirrer, cooler and reaction visualisation system. The synthesis was carried out in temperature-control mode at solvent’s reflux temperature, where the microwave power is regulated by automatic variable-frequency adjustment according to the present temperature and reaction time. The heating rate is continuously monitored and adjusted by feedback control. Significantly, the system operates in a non-pulsed, continuous microwave heating mode. The ATR-FTIR Nicolet iS50 Tri-Detector Gold Flex Spectrometer (Thermo Scientific, Waltham, MA, USA) was employed to monitor the reaction’s course and analyse the obtained products. This spectrometer features a wave number range of 9600–20 cm^−1^ with an interferometer speed of 65 scans/s and separability of 16 cm^−1^. Additionally, the spectrometer is equipped with a fibre optic probe, which connects those two devices, making it a non-invasive and efficient tool for analysis. Integrating these two devices (Figure 9), the synthetic reactor and analytical spectrometer, creates a unique and interdisciplinary research system to effectively study the chemical synthesis processes in real time. The spectral results were obtained and processed via Omnic software (Series 9.12.928). The wavenumber area 1900–600 cm^−1^ was used for analysis in all experiments.

### 3.2. Solvents and Chemicals

Benzil (B), 1,2-phenylenediamine (o-PDA), solvents (EtOH, MEOH, EtOAc, MeCN, DMSO, DCM) and catalyst (HCl, I_2_, Montmorillonite K10) from Aldrich (Saint Louis, MO, USA), Fluka (Buchs, Switzerland), Chempur (Piekary Śląskie, Poland), and POCh S.A. (Gliwice, Poland) were used. All other chemicals of the highest purity were commercially available, and demineralised water was used.

### 3.3. Synthetic Procedures

The preliminary research involved the condensation of benzil (1,2-diphenylethane-1,2-dione) (B) and 1,2-phenylenediamine (o-PDA)—3,2 mmol each with a catalyst to produce 2,3-diphenylquinoxaline (2,3-diphenyl-benzopyrazine) (2,3-DPQ) (Figure 2). Different solvents were chosen for the kinetics evaluation: methanol (MeOH), ethanol (EtOH), dimethyl sulfoxide (DMSO), methanol–water (7:3), acetonitrile (MeCN), ethyl acetate (EtOAc), water, dichloromethane (DCM). Reagents and solvents were purchased from commercial sources.

#### General Procedure of 2,3-DPQ Synthesis

(1) MW method: A total of 10 mL of the selected solvent was placed in a reaction flask along with the catalyst, which consisted of 5 drops of concentrated hydrochloric acid (HCl) or 1 mL of 10% HCl aqueous solution or 5–10 mol% I_2_ or 10–40 mol% of Montmorillonite K10. The background spectra of the solvent and catalyst were recorded before adding reactants. Subsequently, 3.2 mmol (0.673 g, 1.0 eq) of B was introduced into the reaction flask containing the solvent and catalyst. The mixture was heated to facilitate the dissolution of B by microwave irradiation at either 100 W or 200 W power settings. Spectral data collection was initiated, and after 1.5–2 min of spectra recording, 3.2 mmol (0.346 g, 1.0 eq) of o-PDA was added to the reaction mixture. The reaction was allowed to proceed under continued microwave irradiation until completion (full conversion) under reflux, as monitored by in situ FTIR spectral analysis and TLC. Full conversion was not observed in DMSO, MeOH–Water and Water.
(2) Conventional heating: A total of 10 mL of the selected solvent was placed in a reaction flask along with the catalyst, which consisted of either five drops of concentrated hydrochloric acid (HCl) or 1 mL of 10% HCl aqueous solution or 5–10 mol% I_2_ or 10–40 mol% of Montmorillonite K10. The background spectra of the solvent and catalyst were recorded before adding reactants. Subsequently, 3.2 mmol (0.673 g, 1.0 eq) of B was introduced into the reaction flask containing the solvent and catalyst. The mixture was heated to facilitate the dissolution of B. Spectral data collection was initiated. After 1.5–2 min of spectra recording, 3.2 mmol (0.346 g, 1.0 eq) of o-PDA was added to the reaction mixture. The reaction was allowed to proceed under continued heating until completion under reflux, as monitored by in situ FTIR spectral analysis (presented in Supplementary Materials) and TLC (after the finishing of MW irradiation). Complete conversion was not observed in DMSO, MeOH–Water and Water.

Upon completion, the reaction mixture was cooled to room temperature, and the resulting product was isolated by filtration. The crude product was then purified by column chromatography using chloroform and methanol (15:1, *v*/*v*) as the eluent and Silica gel 60 (63–200 μm particle size, Merck was used. The final product was characterised using standard analytical techniques, such as melting point determination, ex situ FTIR, nuclear magnetic resonance (NMR) spectroscopy, and mass spectrometry (MS) to confirm its structure and purity. The ^1^H and ^13^C NMR spectra were recorded using an NMR Varian VNMR-S 400 MHz spectrometer at 400 and 100 MHz, respectively (Agilent Technologies, Santa Clara, CA, USA). The chemical shifts were expressed in parts per million (ppm) relative to tetramethylsilane (TMS) as an internal standard, using CDCl_3_ as the solvent. The MS spectra were recorded on a Bruker 320MS/420GC spectrometer apparatus (Bruker Corporation, Billerica, MA, USA) using the electron impact technique (EI), operating at 75 eV. Collected data about purity were in full accordance with literature information [26,35,36] and presented in the Supplementary Materials. The reaction mixture was also checked using the TLC method on silica gel plates (DC-Alufolien Kieselgel 60 F254 from Merck, Darmstadt, Germany). The TLC spots on the plates were observed in UV light (λ = 254 nm).

### 3.4. Analysis Setup Procedures

During the experimental procedure, the progress of the reaction was monitored in real time using IR spectroscopy, with time-resolved measurements (32–64 scans) conducted for each solvent under different conditions: MW 100 W, MW 200 W, and conventional reflux. Before all experiments, a solvent + catalyst system background was obtained. Additionally, the reaction progress was monitored using the TLC method to confirm the completion of the reaction. The intensity profiles of selected characteristic peaks were meticulously recorded and archived for analysis. For better comparability between different solvents and more straightforward interpretation of the results, normalised intensity was calculated using min–max normalisation, where the minimum intensity of each spectrum was set to 0 and the maximum to 1. The reaction’s endpoint was determined by observing the stabilisation and the value of peak intensities. Each experiment was repeated at least five times for every solvent–condition combination, and the results were evaluated for reproducibility.

## 4. Conclusions

This study successfully tested, characterised, and demonstrated the efficacy of a novel method of coupling in situ FTIR spectroscopy with MW irradiation for organic synthesis and highlighted its potential as a powerful tool, particularly in the context of heterocyclisation reactions. While both techniques are well established individually, their combination remains largely unexplored. Our findings show how this integrated approach can be used in routine synthesis, offering enhanced reaction monitoring, improved reproducibility, and real-time process control—key factors for modern chemical and pharmaceutical manufacturing.

The research identified the optimal pathway for synthesising 2,3-diphenylquinoxaline through condensation of diketone and diamine under MW (200 W) irradiation, using MeCN or EtOAc as solvent and molecular iodine (5 mol%) as catalyst. Raising the reaction temperature from room temperature to reflux significantly shortened the reaction time for all solvent variants. Increasing microwave power from 100 to 200 W further reduced the reaction time and increased the yield. Importantly, several solvents and catalysts often reported in the literature as promising for similar transformations were also tested here. However, careful re-evaluation with IR spectroscopy and complementary analytical methods revealed them to be inefficient and ultimately unsuitable for this reaction. This finding underlines the need for experimental validation and shows that published conditions cannot always be applied directly, particularly when aiming to optimise reactions for efficiency and reproducibility.

Building on these findings, our research will expand this approach to synthesise other nitrogen-heterocyclic compounds relevant to the pharmaceutical industry. Future work will focus on adapting the method for larger-scale reactions, assessing its compatibility with different reaction classes, and further optimising its parameters for efficiency and sustainability. Refining this methodology aims to enhance its practical applicability for potential industrial use and provide valuable insights into developing efficient, scalable synthetic routes for bioactive compounds.

## Figures and Tables

**Table 1:** Characteristic FTIR peaks for chemical structures of the reagents and products.

Compound	Peak (cm^−1^) by Ex Situ FTIR	Characteristic Group	Literature Source
B	3050	=C–H stretch from the phenyl rings	[28,29]
	1656	C=O stretching of the diketone	[28,29]
	1591, 1577	aromatic C=C in-ring stretches (phenyl moieties)	[28,29]
	1453	aromatic C–C stretching or C-H in-plane bending	[28,29]
	1209	C-C bend. of ketones/C–C stretches in the phenyl ring	[28]
	1173	C-C bend. of ketones/C–C stretches in the phenyl ring	[28]
	874, 716, 679	Out-of-plane C–H bending modes of monosubstituted benzene rings	[28]
o-PDA	3384, 3183	N–H stretching vibrations of the primary amine	[29]
	3027	Aromatic C–H stretching of the benzene ring	[29,30]
	1636	N–H bending (scissoring) in primary amines	[29,30]
	1590, 1272, 1247	C–N stretching vibrations of aromatic amines and may overlap with in-plane N–H bending or ring deformation modes	[29,30]
	744	out-of-plane C–H bending of ortho-substituted benzene	[29,30]
2,3-DPQ	3057	Aromatic =C–H stretch (sp^2^ C–H)	[29,31,32,33]
	1558, 1541	C=N stretching of quinoxaline conjugated with aromatic C=C stretch	[33]
	1442	C-C=N/C=C	[32,33]
	1346	Aromatic C–N stretch or CH in-plane bend	[29,31,33]
	1219	C-N/in-plane CH bending region	[29,31,32]
	1142	C-N/in-plane CH bending region	[29,31]
	977	C-H	[29]
	762	out-of-plane =C–H bending for monosubstituted phenyl rings	[29,31,32,33]
	694	C-H (plane vibrations of the arom. ring)	[29,31]
	596, 564	C-C-N (skeletal ring deformation modes)	[31]

**Table 2:** Reaction mixture at the completion of reaction—in situ peaks analysis in MeOH.

Peak (and Peak’s Borders) by In Situ FTIR, cm^−1^	Functional Group	From Which Compound
1636 (1694; 1583)	O-H	H_2_O
1442 (1452; 1426)	C-C=N/C=C	DPQ
1345 (1358; 1295)	C-N/C-C(phenyl)	DPQ
1225 (1252; 1200)	C-N/C-H	DPQ
1149 (1163; 1120)	C-N/C-H	DPQ
809 (823; 792)	C-H (plane vibrations of the aromatic ring)	DPQ
772 (792; 739)	C-H (monosubstituted phenyl ring)	DPQ
700 (718; 677)	C-H (aromatic ring)	DPQ

**Table 3:** Average time and isolated yield of reaction completion in different solvents and conditions with concentrated HCl and I_2_ 5 mol% as catalyst.

Condition/Solvent	MW 200 W			MW 100 W			Reflux			Stirring at Room Temperature	
HCl conc.											
	B Consumption Time [min]	2,3-DPQ PGS * [min]	Yield *** [%]	B Consumption time [min]	2,3-DPQ PGS * [min]	Yield *** [%]	B Consumption time [min]	2,3-DPQ PGS * [min]	Yield *** [%]	Approximate Reaction Finish Time [h]	Yield *** [%]
MeCN	3	10	98	7	22	97	18	31	93	2	89
EtOH	16	36	96	25	54	95	43	80	91	3.2	86
MeOH	23	42	96	36	58	95	52	102	91	5	86
I_2_ 5 mol%											
MeCN	3.5	6	99	6	8	98	9.5	11	95	40 min	91
EtOAc	3.5	6	98	6	8	97	13	17.5	93	45 min	89
EtOH	30	45	95	- **	- **	- **	68	80	91	3.5	88
MeOH	40	56	95	- **	- **	- **	130	190	90	7.5	87

* PGS—peak growth stabilisation time—in min; **—since under 200 W microwave irradiation in MeOH and EtOH the reaction required more than 45 min, which is suboptimal for microwave synthesis, the measurement at 100 W was not performed; ***—isolated yield by column chromatography.

**Table 4:** Time reduction factor for MeCN with HCl and I_2_ 5 mol% as catalyst.

	Reaction Time [min]			Time reduction Factor in Different Reaction Conditions
	b.p./Reflux	MW-100 W	MW-200 W	
HCl	31	22	10	3:2:1
I_2_	11	8	6	2:1.3:1
Time reduction overall factor in different solvents	1:3	1:3	1:2	

**Table 5:** Difference in reaction time between I_2_ at 10mol% and 5 mol% as catalyst.

	I_2_ Load at Room Temperature Mixing	
	5 mol%	10 mol%
MeCN	45 min	4 h
EtOH	3.5 h	Not complete
MeOH	7.5 h	Not complete

## Data Availability

Data are contained within the article and Supplementary Materials.

## Supplementary Materials

The following supporting information can be downloaded at: https://www.mdpi.com/article/10.3390/molecules30193875/s1, Figure S1: 3D in situ FTIR spectra and heatmap of the 2,3-diphenylquinoxaline synthesis at 200W MW for I_2_ 5mol% as catalyst; Figure S2: 3D in situ FTIR spectra and heatmap of the 2,3-diphenylquinoxaline synthesis in different solvent/catalyst systems at 200W MW for HCl_conc_ as catalyst; Figure S3: NMR data of the product—2,3−diphenylquinoxaline; Figure S4: Mass spectra of the product—2,3-diphenylquinoxaline; Figure S5: Ex situ ATR-FTIR spectra of 2,3-Diphenylquinoxaline.
