# Supplementary material for: A Novel Method of Coupling In Situ Time-Resolved FTIR and Microwave Irradiation: Application to the Monitoring of Quinoxaline Derivatives Synthesis
Source: Molecules. 2025 Sep 25;30(19):3875. doi: 10.3390/molecules30193875 (PMC12525907; doi:10.3390/molecules30193875)

Supplementary Material

A Novel Method of Coupling In Situ Time-Resolved FTIR and Microwave Irradiation: Application to the Monitoring of Quinoxaline Derivatives Synthesis

Alina Cherniienko, Kacper Kossakowski, Lucjusz Zaprutko, Roman Lesyk, Dorota Olender and Anna Pawełczyk

Figure S1. 3D in situ FTIR spectra and heatmap of the 2,3-diphenylquinoxaline (2,3-DPQ) synthesis at 200W MW for I_2 5mol% as catalyst:

a) MeCN – 200W MW, I_2 5mol% as catalyst: 2,3-DPQ peak growth stabilisation time - 6 min.

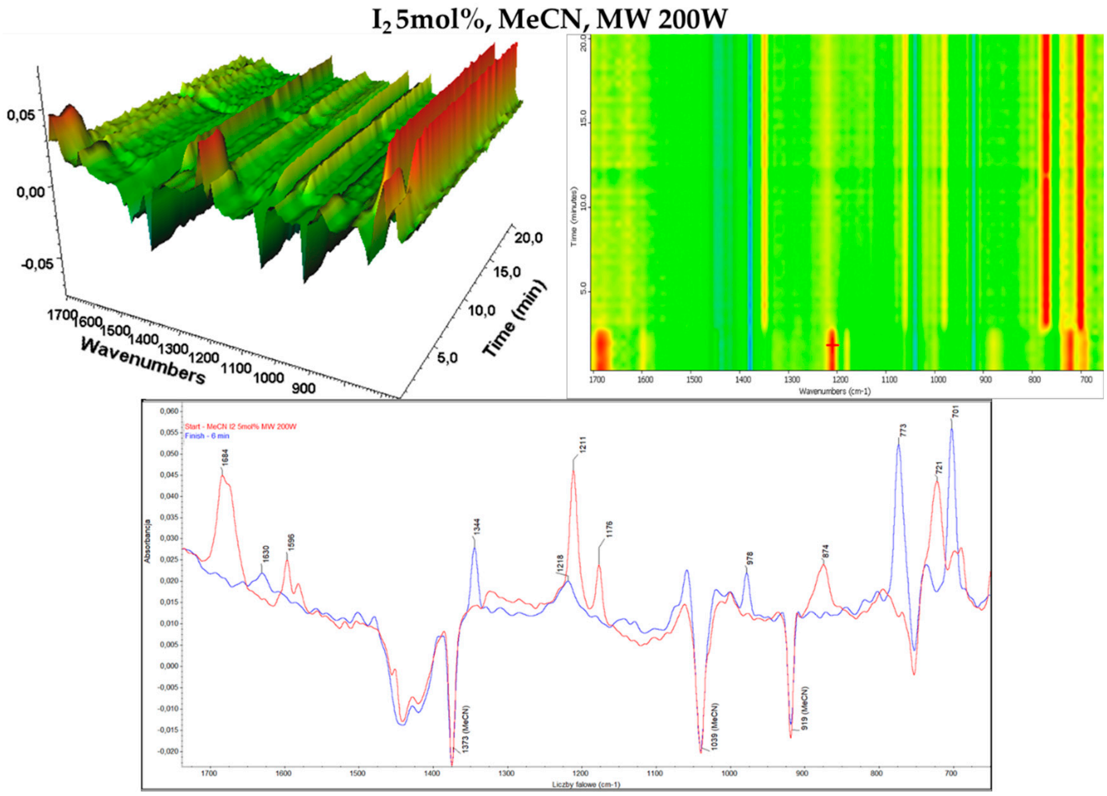

b) EtOAc – 200W MW, I2 5mol% as catalyst: 2,3-DPQ peak growth stabilisation time - 6 min.

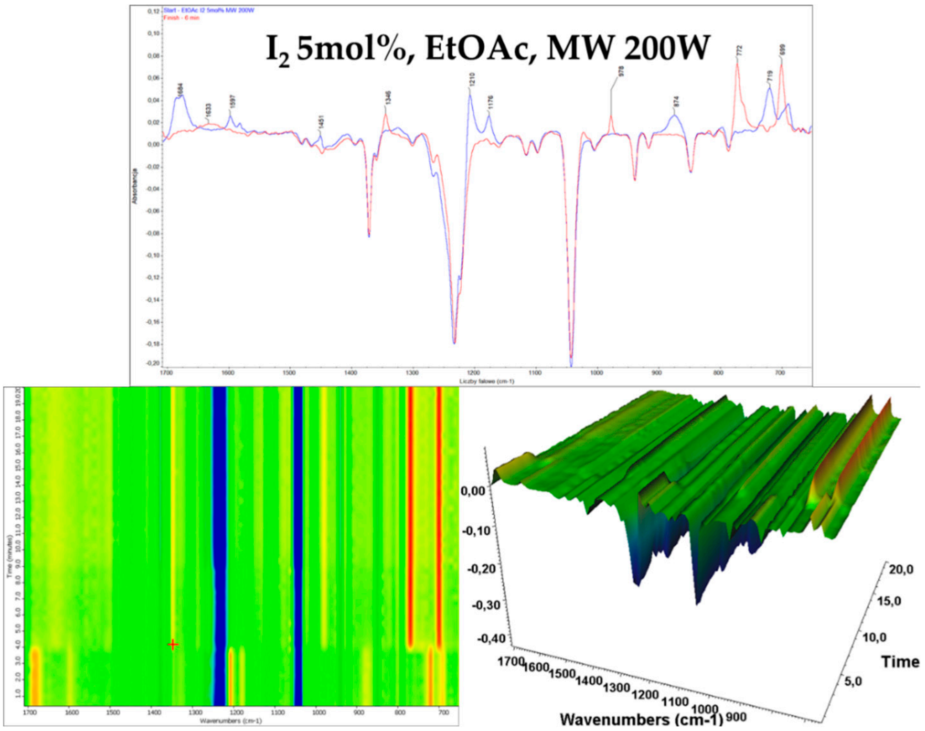

c) EtOH – 200W MW, I2 5mol% as catalyst: 2,3-DPQ peak growth stabilisation time - 45 min.

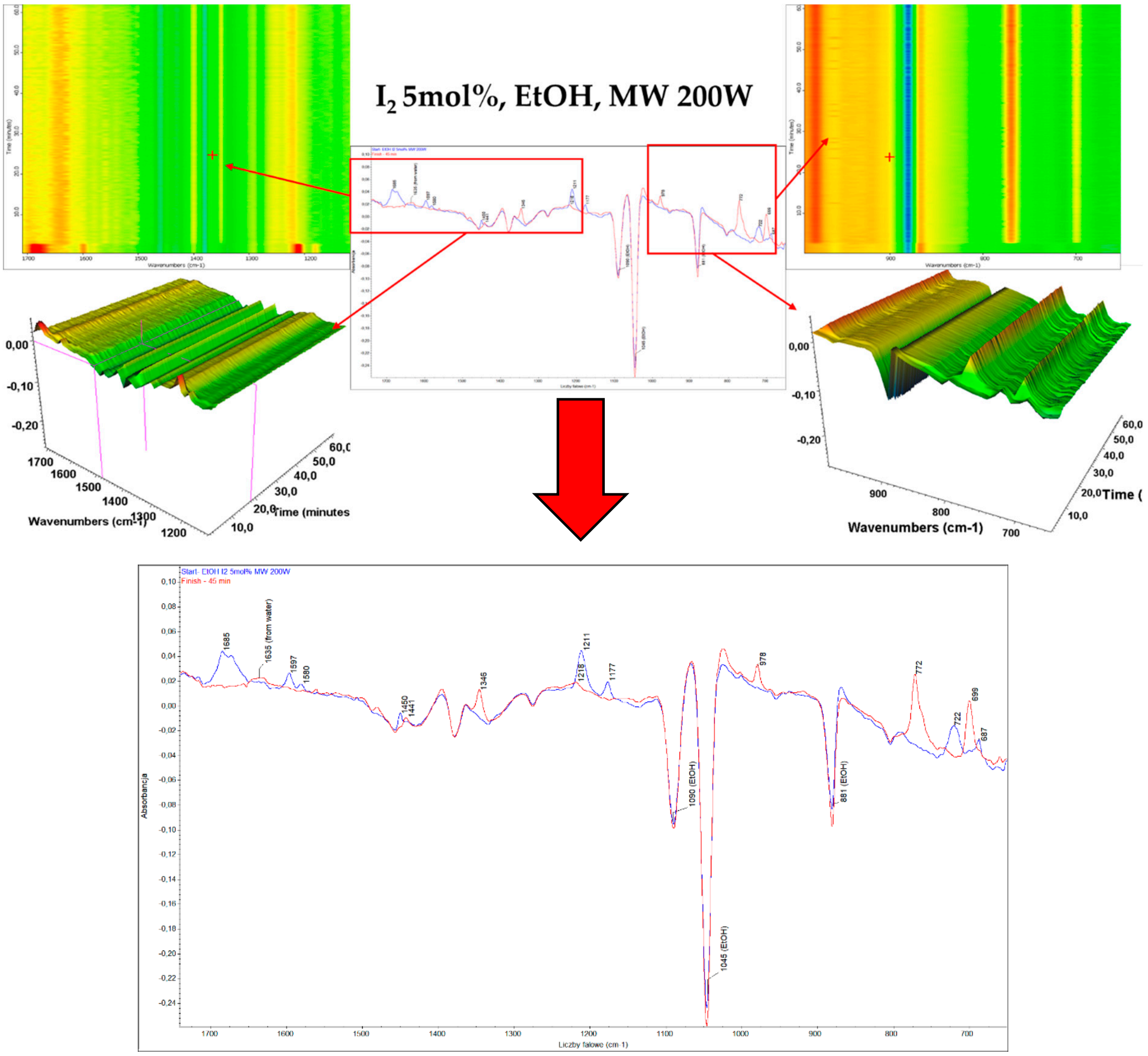

d) MeOH – 200W MW, I2 5mol% as catalyst: 2,3-DPQ peak growth stabilisation time - 56 min.

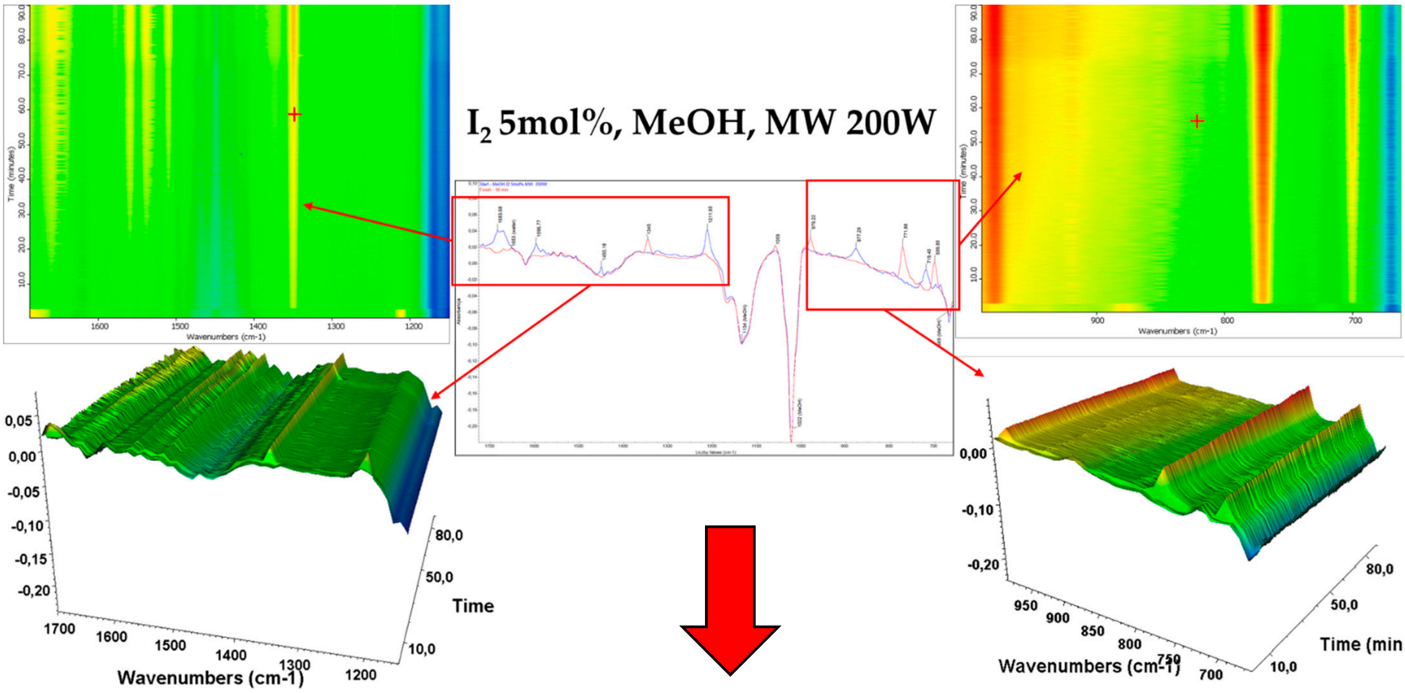

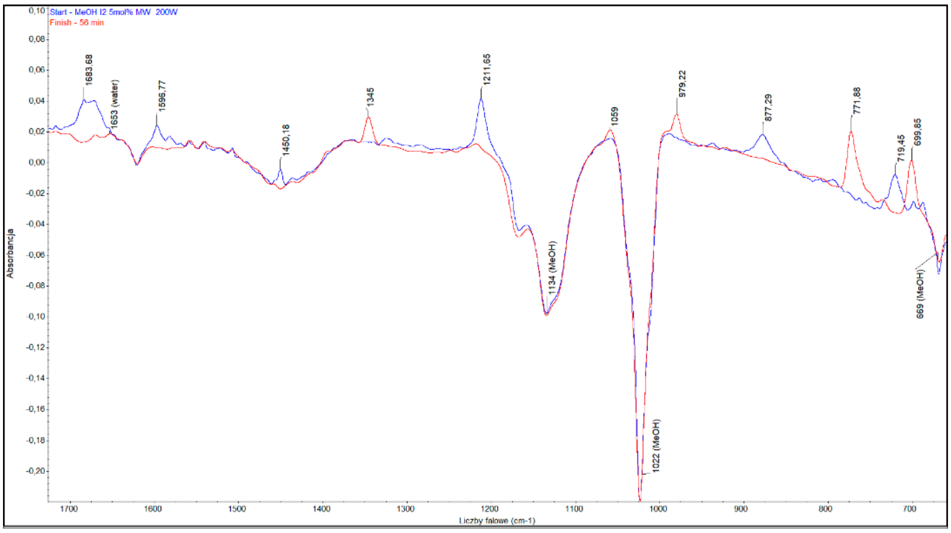

Figure S2. 3D *in situ* FTIR spectra and heatmap of the 2,3-diphenylquinoxaline synthesis in different solvent/catalyst systems at 200W MW for HCl_{conc} as catalyst:

a) MeCN – 200W MW, HCl_{conc} as catalyst: 2,3-DPQ peak growth stabilisation time - 10 min.

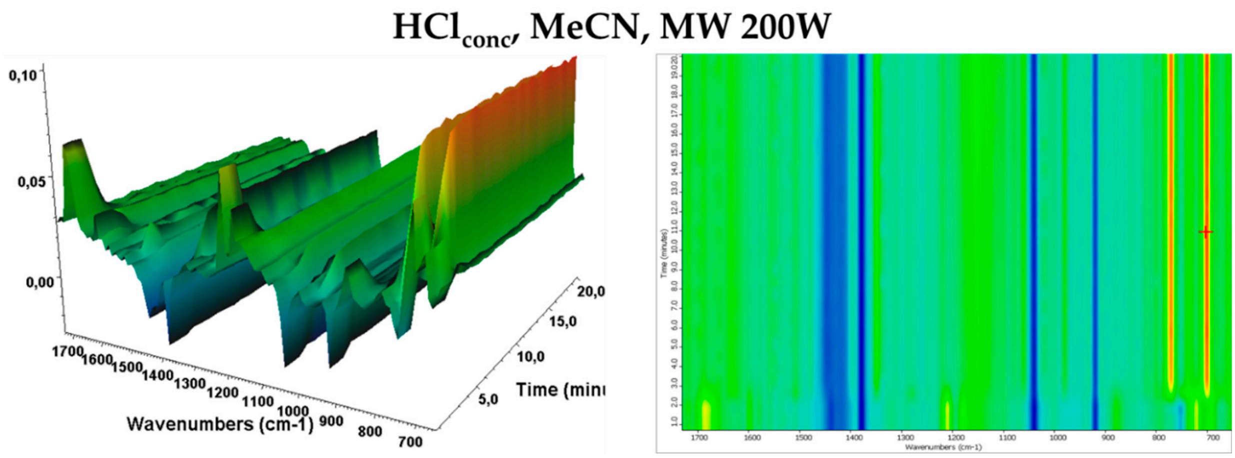

b) EtOH – 200W MW, HCl_{conc} as catalyst: 2,3-DPQ peak growth stabilisation time - 36 min.

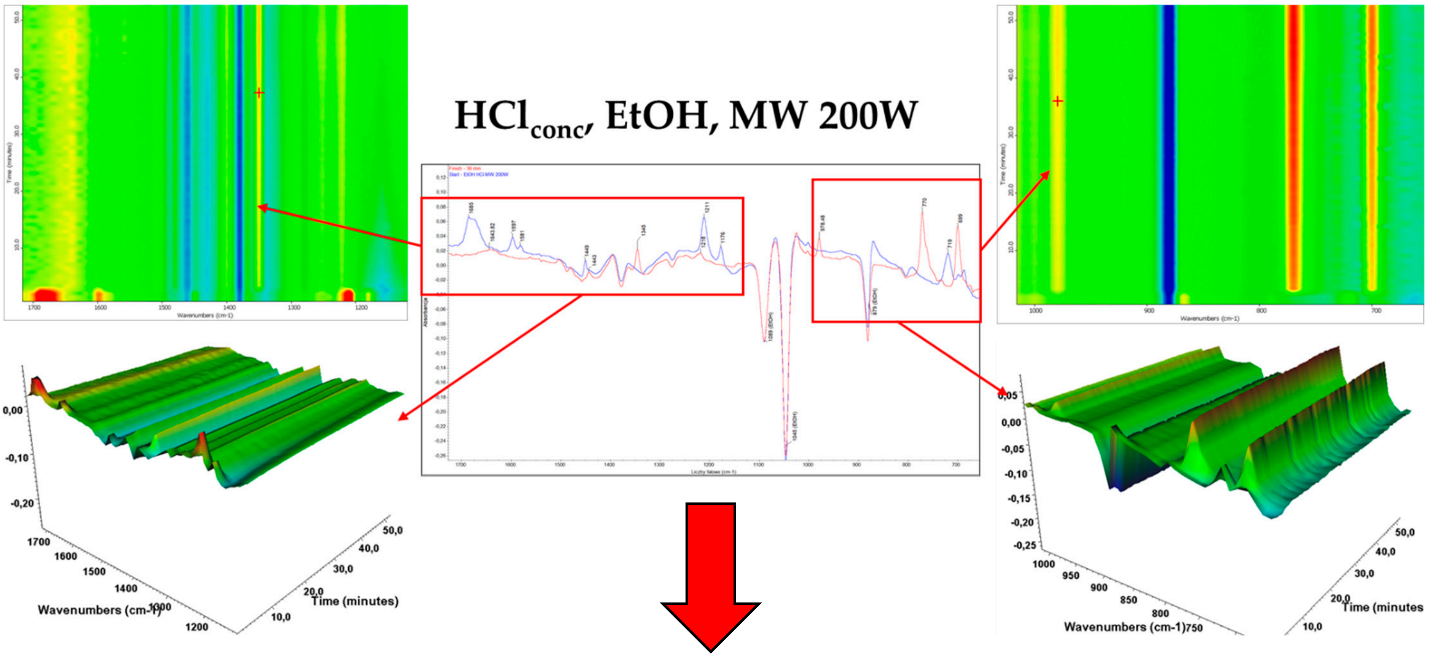

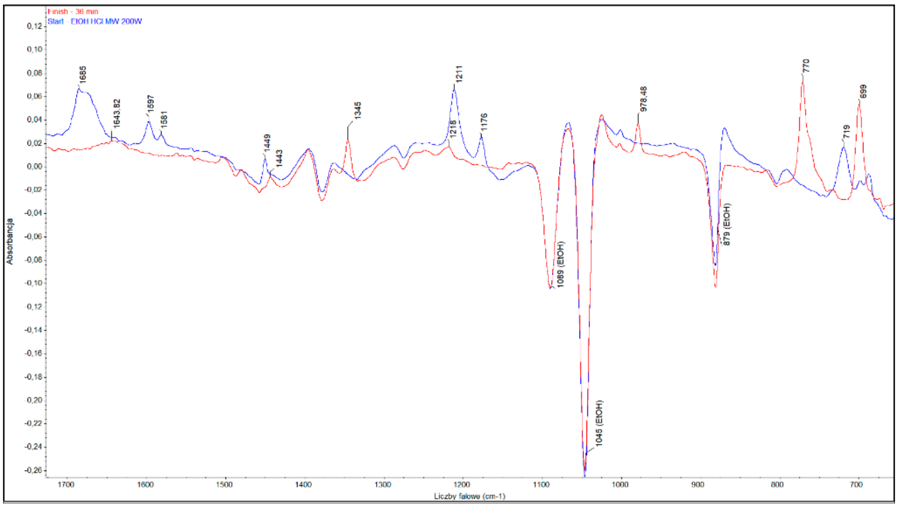

c) **MeOH – 200W MW, HClconc as catalyst: 2,3-DPQ peak growth stabilisation time - 42 min.**

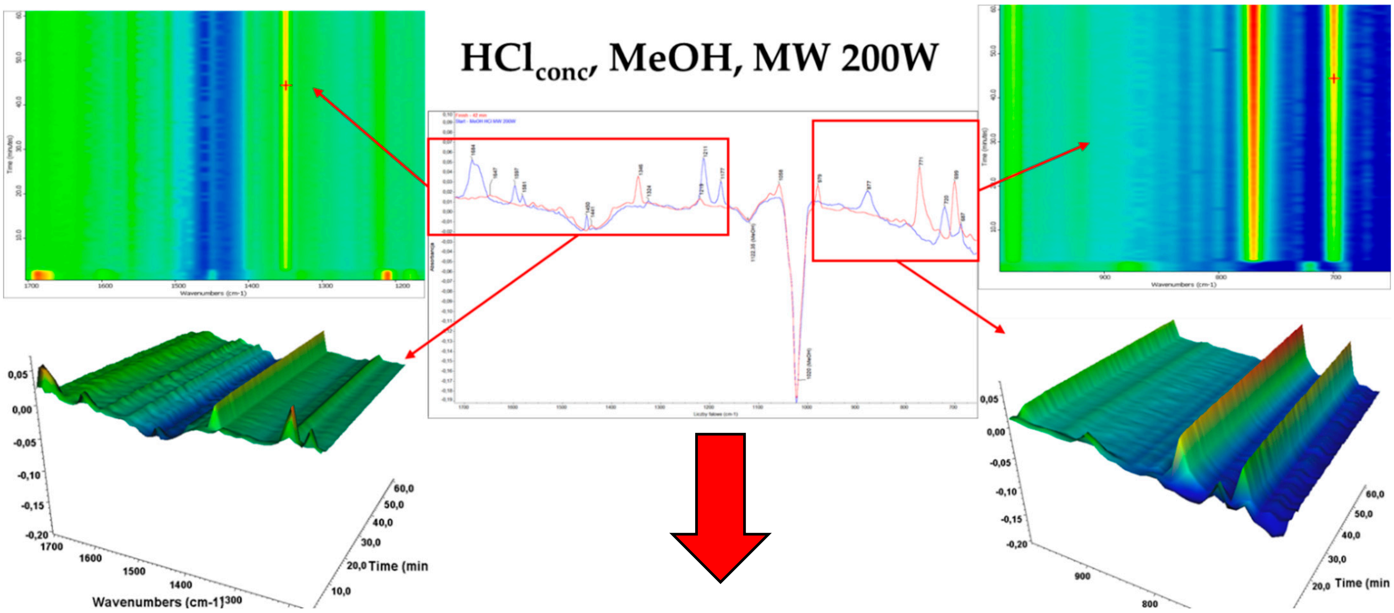

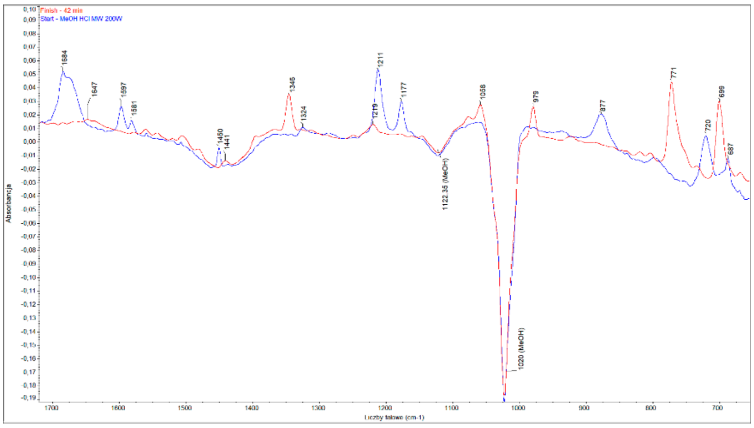

Figure S3. NMR data of the product – 2,3-diphenylquinoxaline

a) **^{1}H NMR** ($CDCl_3$, 400 MHz,) δ 8.24 – 8.19 (2H, m), 7.82 – 7.78 (2H, m), 7.58 – 7.53 (4H, m), 7.40 – 7.34 (6H, m)

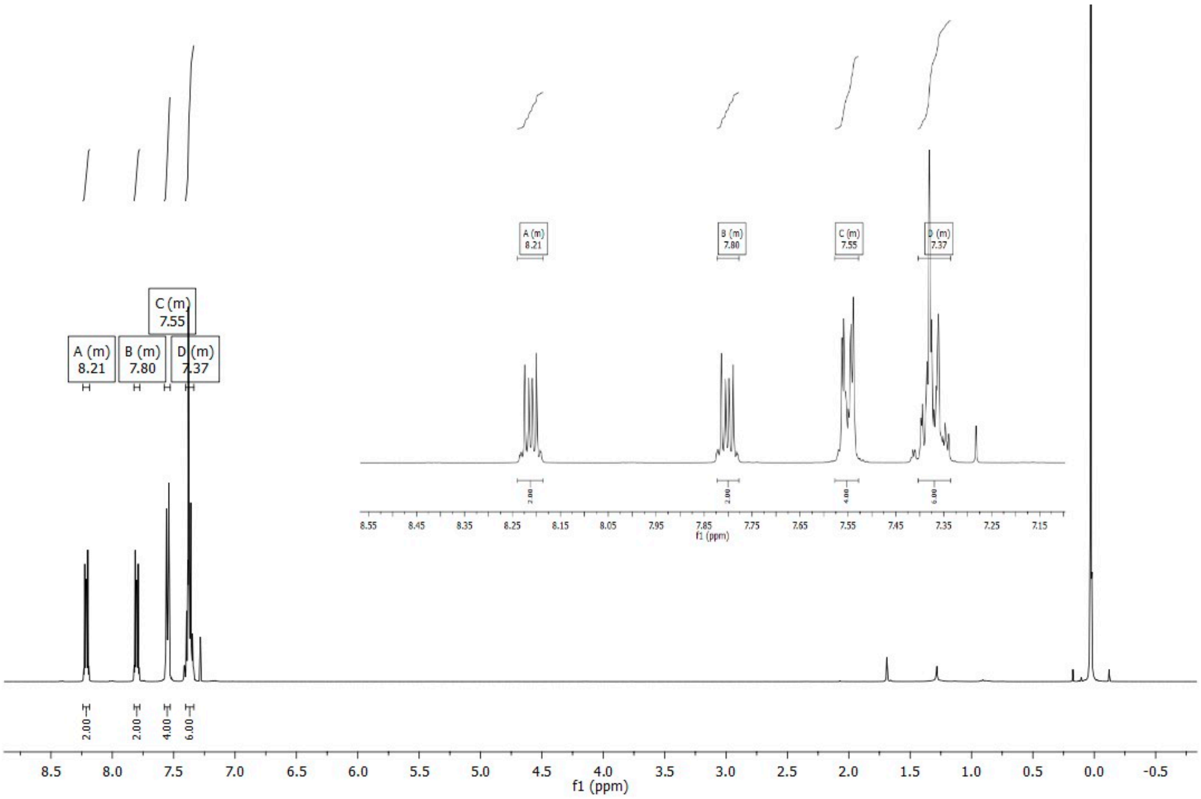

b) **^{13}CNMR** ($CDCl_3$, 101 MHz,) δ 153.50 (2C, s), 141.26 (2C, s), 139.10 (2C, s), 129.99 (2C, s), 129.86 (4C, s), 129.24 (2C, s), 128.83 (2C, s), 128.30 (4C, s).

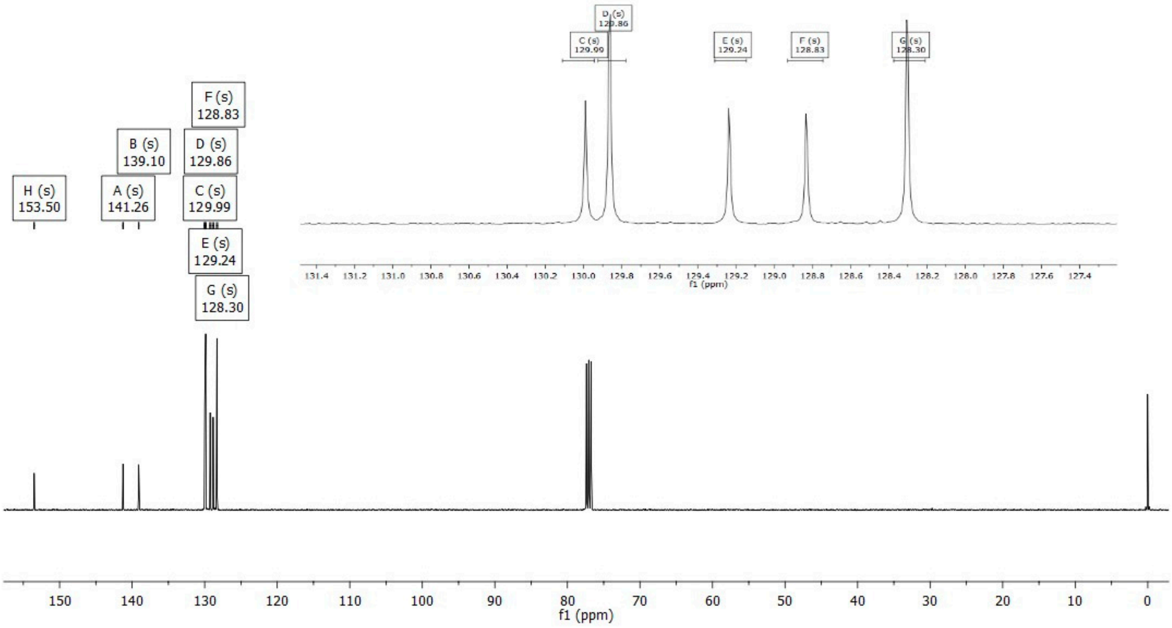

Figure S4. Mass spectra of the product - 2,3-diphenylquinoxaline

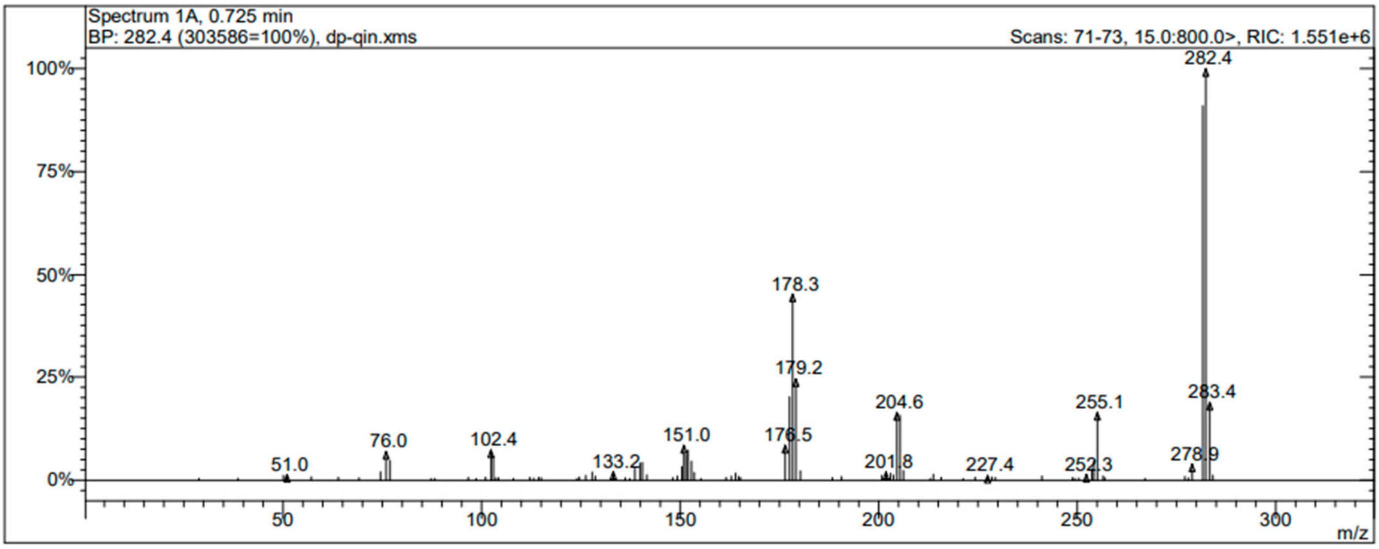

Figure S5. Ex situ ATR-FTIR spectra of 2,3-Diphenylquinoxaline.

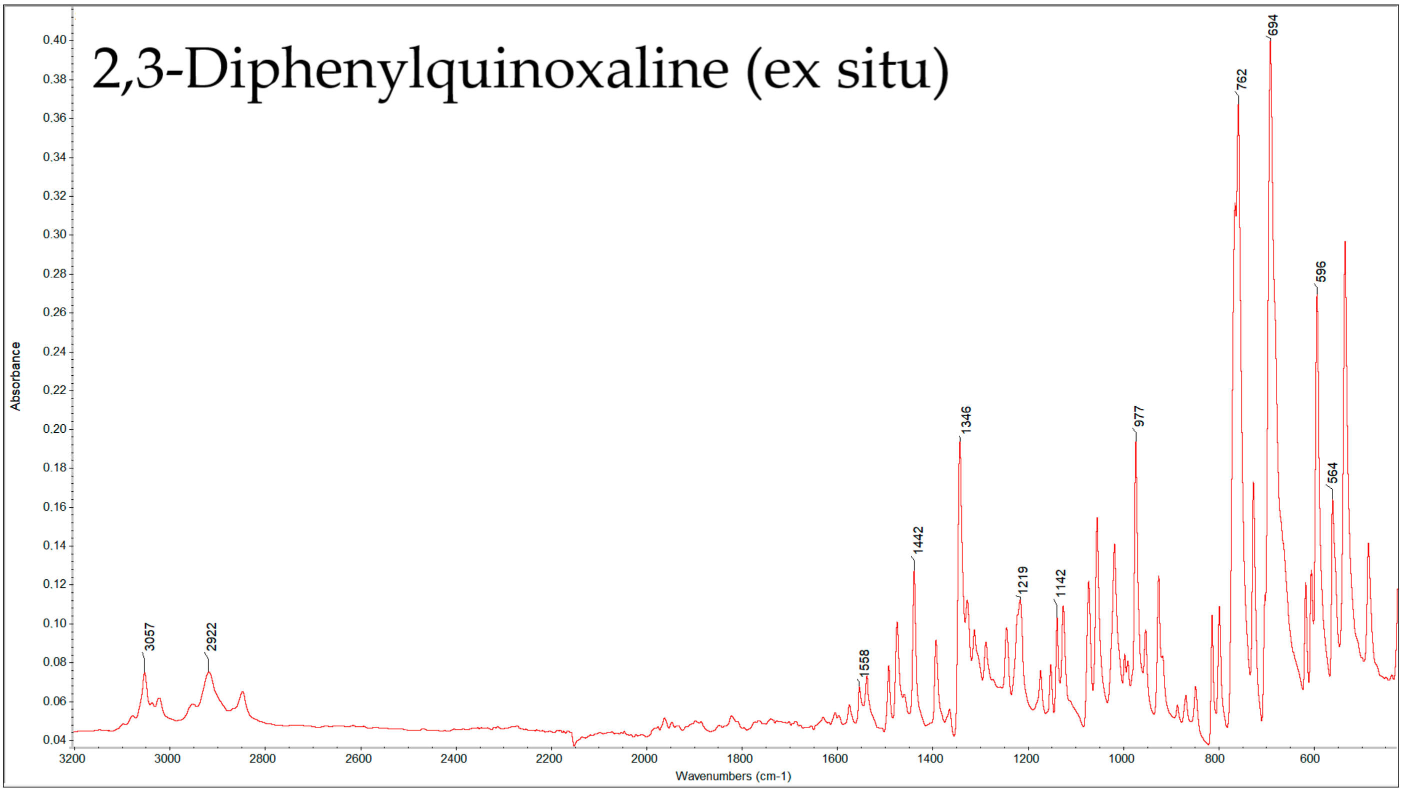

Supplement: Supplementary file 1 [file molecules-30-03875-s001.zip › molecules-3867980-supplementary.pdf]
